# The Relationship Between Body Mass Index and Recurrence Risk of Stroke: A Systematic Review and Dose–Response Meta‑Analysis

**DOI:** 10.1002/brb3.70550

**Published:** 2025-05-29

**Authors:** Qiuxia Qian, Yuting Zhao, Xin Fan, Jialu Li, Jianxun Cao, Mengyu Yang, Longchun Hua, Xingxia Zhang, Ailing Yang, Fengwa Zhang, Yuxia Ma

**Affiliations:** ^1^ Evidence‐Based Nursing Center, School of Nursing Lanzhou University Lanzhou China; ^2^ The First Hospital, Lanzhou University Lanzhou Gansu China; ^3^ Department of Radiology Gansu Provincial Hospital Lanzhou Gansu China; ^4^ Department of Digestive Endoscopy Gansu Provincial Hospital Lanzhou Gansu China; ^5^ Operating Room Gansu Third People's Hospital Lanzhou Gansu China; ^6^ The Second Hospital, Lanzhou University Lanzhou Gansu China; ^7^ Burns Surgery/Plastic Surgery Gansu Provincial Hospital Lanzhou Gansu China

**Keywords:** body mass index, dose–response analysis, meta‐analysis, stroke recurrence

## Abstract

**Objective:**

To explore the relationship between body mass index (BMI) and the recurrence risk of stroke.

**Methods:**

We searched databases, including the Web of Science, Cochrane Library, Embase, PubMed, Chinese Biomedical Literature (CBM), CQVIP, WanFang Database, and China National Knowledge Infrastructure (CNKI), from inception to February 2025, to collect literature on BMI and the recurrence risk of stroke. After two researchers independently screened the literature, extracted the literature data, and assessed the quality of the literature included in the study, a meta‐analysis was conducted using Stata 16.0 software, and the dose–response relationship between BMI and the recurrence risk of stroke was analyzed using generalized least squares trend estimation method (GLST) and restricted cubic spline function.

**Results:**

A total of 18 studies were included, involving 165,366 patients. In terms of stroke recurrence risk, compared with normal‐weight patients, underweight patients [relative risk (RR) = 1.59, 95% confidence interval (CI) 1.33–1.90, *I* ^2^ = 0%, *p *= 0.444] had a higher recurrence risk of stroke, whereas overweight (RR = 0.91, 95% CI 0.86–0.96, *I* ^2^ = 0%, *p *= 0.454) and obese patients (RR = 0.89, 95% CI 0.84–0.94, *I* ^2^ = 13.1%, *p *= 0.330) had a lower recurrence risk of stroke. The results of the linear trend show that for every unit increase in BMI, the recurrence risk of stroke decreases by 2% (RR = 0.98, 95% CI 0.96–0.99, *p *< 0.001).

**Conclusion:**

Increased BMI is associated with a decreased recurrence risk of stroke. Underweight is a risk factor for stroke recurrence, whereas overweight and obesity are protective factors for stroke recurrence. Overweight and obesity may be beneficial for secondary prevention in stroke patients.

**Clinical Trial Registration:**

Not applicable.

## Graphical abstract

The obesity paradox can be extended to stroke recurrence. A 2% reduction in recurrence risk of stroke per 1‐unit increase in BMI.

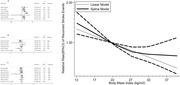

## 1 Introduction

Stroke is defined as an episode of acute neurological dysfunction presumed to be caused by ischemia or hemorrhage, persisting for more than 24 h or until death (Hankey 2017). Stroke is characterized by a high incidence rate, high disability rate, high mortality, high recurrence rate, and high economic burden (Longde et al. 2022). The Global Burden of Diseases 2019 (GBD) study suggested stroke remained the second‐leading cause of death and the third‐leading cause of death and disability globally (Feigin et al. 2021). From 1990 to 2019, the burden of stroke significantly increased, with a 70.0% increase in stroke incidence, a 43.0% increase in stroke deaths, and a 102.0% increase in stroke prevalence (Feigin et al. 2021). It is estimated that the global losses caused by stroke exceed 891 billion US dollars, accounting for 1.12% of global GDP (Owolabi et al. 2022). However, stroke recurrence is the main cause of long‐term disability and increased mortality in patients (Gao et al. 2020).

Recurrent stroke is defined as the appearance of new neurological deficits after a clinically stable period, lasting for more than 24 h, and confirmed by CT or MRI to be attributed to new ischemic or hemorrhagic lesions (Kolmos et al. 2021). The recurrence risk of stroke at 30 days, 1 year, 5 years, 10 years, and 12 years after the first stroke was 3.1%, 11.1%, 26.4%, 39.2%, and 39.7%, respectively (Lin et al. 2021; Mohan et al. 2011). Compared with the first stroke, the stroke recurrence is more disabling, fatal, and costly, with a 43% higher risk of death and prolonged length of stay, which seriously affects the life quality of patients (Lekoubou et al. 2017; Samsa et al. 1999), increasing the burden on caregivers and the economic burden on families, society, and the country. Therefore, reducing the recurrence risk of stroke is very important and has the significance of reducing the burden on society, the country, and families.

There are many risk factors for stroke recurrence, including both modifiable (e.g., physical exercise, body mass index [BMI], diet, smoking, drinking, comorbid conditions) and nonmodifiable risk factors (e.g., age, sex, race) (Boehme et al. 2017). About 90% of strokes are caused by the presence of modifiable risk factors, and the regulation of core health behaviors could avert about 75% of this burden (Feigin et al. 2016; Tsao et al. 2022). Thus, the key to reducing stroke recurrence risk is to identify modifiable risk factors.

There is overall agreement in the scientific and clinical community that obesity is one of the major public health problems and is increasingly recognized as an important risk factor for recurrent stroke (Flegal et al. 2012; Mitchell et al. 2015; Pezzini et al. 2013; Prospective Studies 2009; Winter et al. 2008). However, BMI is not only an important indicator for determining the degree of obesity and thinness in the human body (Hao and Wenkai 2001), but also a cheap and convenient method for screening weight categories, providing a simple and feasible intervention measure for stroke recurrence.

Recent studies (Olsen et al. 2008; Vemmos et al. 2011) reported that the stroke obesity paradox may even be extended to include the recurrence risk of stroke. One study (W. Chen et al. 2019) has reported that overweight patients were at higher recurrence risk of stroke than those of normal weight, but no significant relationship was observed in obese patients, but the other study (Andersen and Olsen 2013) indicated that obese patients had a lower risk and underweight patients had a higher recurrence risk of stroke, so the relationship between BMI and recurrence risk of stroke is unclear. So far, only one study (Huang et al. 2016) has included relevant cohort studies before December 15, 2015 to conduct a systematic evaluation and dose–response meta‐analysis of the relationship between BMI and recurrence risk of stroke, which needs to be updated. Therefore, we performed a systematic review and dose–response meta‐analysis to examine the relationship between BMI and the recurrence risk of stroke, intending to find controllable factors of stroke recurrence to decrease its recurrence risk, thus improving the quality of life of patients and reducing the economic burden of family, society, and country.

## 2 Methods

The present study was performed according to Preferred Reporting Items for Systematic Reviews and Meta‐Analyses (PRISMA‐2009) guidelines (Liberati et al. 2009). The detailed study protocol can be obtained on the PROSPERO website with the registration number CRD42023447236. On the basis of the information provided in the study protocol, we performed a dose–response analysis, and the search date was extended to February 2025.

### 2.1 Data Sources and Search Strategy

Comprehensive literature searching was conducted in PubMed, Embase, Web of Science, Cochrane Library, CQVIP, WanFang Database, China National Knowledge Infrastructure (CNKI), and Chinese Biomedical Literature (CBM) from inception to February 2025. Additionally, reference lists included in the identified articles were manually searched to identify additional relevant publications. We performed the search strategy through a combination of MeSH terms and free words. The specific search strategies can be found in the appendix, and the PubMed search strategy is provided as an example; the search details are provided in Table S1.

### 2.2 Study Selection

Two researchers were asked to perform literature screening independently. Literature retrieved from the initial database search was screened for duplicates and excluded before other exclusion procedures. Full‐text articles were retrieved when at least one reviewer decided that an abstract was eligible for inclusion. Each publication was assessed independently by both researchers for final study inclusion. Disagreements were resolved by discussion with the third researcher.

The inclusion criteria were as follows: ① Case–control, cross‐sectional, or cohort studies; ② study subjects were patients with a definite diagnosis of stroke; ③ the study analyzed the relationship between BMI and recurrence risk of stroke; ④ the BMI grouping criteria were based on WHO standards (Obesity: preventing and managing the global epidemic. Report of a WHO consultation 2000): <18.5 kg/m^2^ for underweight, 18.5–24.9 kg/m^2^ for normal weight, 24.9–29.9 kg/m^2^ for overweight, and ⩾30 kg/m^2^ for obesity; ⑤ stroke recurrence confirmed by CT or MRI. The exclusion criteria were as follows: ① case reports, reviews, letters, and comments; ② the language of the publication was other than English or Chinese; ③ studies contained incomplete data.

### 2.3 Data Extraction and Quality Assessment

Relevant data from the included studies were extracted independently by two researchers, and the results were cross‐checked. Any disagreements were resolved by discussion with the third researcher. The following information was recorded: first author name, publication year, study location, study design, sample size, gender, age, BMI classification, number of recurrence risk of stroke cases in different BMI groups, and overall number of recurrence risk of stroke cases. All extracted data were stored in the Microsoft Excel file format. The risk of bias assessment of all included studies was conducted using the Newcastle‐Ottawa Scale (NOS) (Stang 2010; Xiantao et al. 2012). We regarded total scores of 0–3, 4–6, and 7–9 as low, moderate, and high quality, respectively. Two researchers independently assessed the quality of the included studies and cross‐checked the results. Any disagreements were resolved by discussion with the third researcher.

### 2.4 Statistical Analysis

The results were quantitatively analyzed using Stata 16.0, using relative risks (RRs) and 95% confidence intervals (CIs) as measures of effect size. We tested heterogeneity among studies using Cochrane's *Q* statistic. The degree of heterogeneity was assessed using the *I* ^2^ statistic, with *I* ^2^ values of 25%, 50%, and 75% being considered to indicate low, moderate, and high heterogeneity, respectively. RRs and 95% CIs were calculated using a random‐effects model when Cochrane's *Q* statistic detected significant heterogeneity; otherwise, a fixed‐effects model was used. The *p* values are the Cochrane's *Q* statistics for the heterogeneity. *p* < 0.05 was the threshold for statistical significance. Sensitivity analysis was conducted using the one‐by‐one elimination method.

A dose–response analysis for BMI and the recurrence risk of stroke was conducted using the generalized least squares trend (GLST) proposed by Orsin and Greenland (Greenland and Longnecker 1992; Orsini et al. 2006) and restricted cubic spline function. The median or mean level of BMI, cases and non‐cases in each BMI group, and corresponding RRs with 95% CIs were collected. The midpoint of each BMI category was utilized if the BMI mean or median for the category was not provided in the study (Rong et al. 2013), which was scientific and to some extent reduced the risk of bias due to missing results in a synthesis. To examine the potential nonlinear associations between BMI and recurrence risk of stroke, we performed a two‐stage random‐effects dose–response meta‐analysis (Orsini et al. 2006).

## 3 Results

### 3.1 Literature Search

**FIGURE 1:**
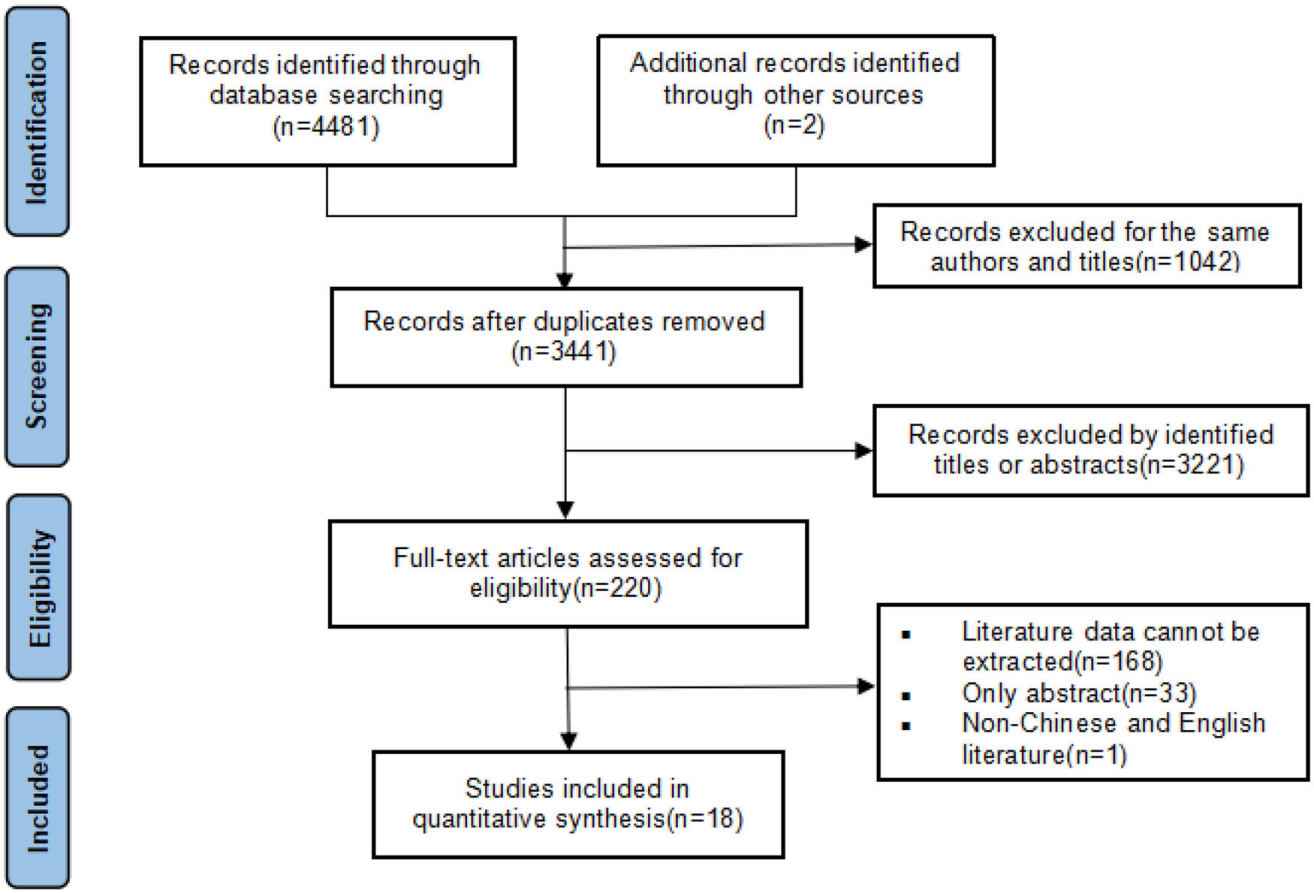
Flow chart for the process of selecting eligible studies.

A total of 4483 relevant literature were obtained through a preliminary search, of which 1042 duplicates were removed. Among the remaining 3441 pieces of literature, 3221 were excluded on the basis of the title and abstract. By reading the full text, 18 studies (Aiming et al. 2014; Andersen and Olsen 2013, 2015; C. Chen et al. 2012; Chung et al. 2023; Doehner et al. 2013; Hou et al. 2021; Hviid Hornnes et al. 2024; Jingguang et al. 2016; Ju et al. 2022; Kawase et al. 2017; Kumral et al. 2021; Olsen et al. 2008; Ovbiagele et al. 2011; Vemmos et al. 2011; Yan et al. 2021; Zhao et al. 2015) met the inclusion criteria and were included. See Figure 1 for the detailed process.

### 3.2 Study Characteristics

We summarized the basic characteristics of the 18 included studies. A total of 18 studies (11 prospective cohort studies, 4 longitudinal studies, and 3 case–control studies) were published between 1997 and 2024 from 8 countries (the United States, Denmark, China, Japan, Korea, Greece, Turkey, and Germany). The sample sizes of the 18 pieces of literature ranged from 100 to 30,912 and contained 165,366 patients. The specific results are shown in Table 1.

**TABLE 1:** The basic characteristics of the included studies.

Study	Year	Country	Sample size	Male (%)	Age (year)	BMI classify (kg/m^2^)	Number of recurrence of stroke in each group	Total number of recurrence of stroke
Hviid Hornnes et al. (2024)	2024	Denmark	1215	—	—	<18.5 18.5–24.9 ≥25	—	406
Jingli et al. (2024)	2024	China	366	62	≥40	<25 ≥25	21 7	28
Chung et al. (2023)	2023	Korea	1584	59.2	Female: 71.9 Male: 67.6	<25 ≥25	489 298	787
Ju et al. (2022)	2022	Korea	30,912	65.7	18–74	<18.5 18.5–24.9	80 1183	1263
Yan et al. (2021)	2021	China	100	60	67.43 ± 3.53	<18.5 ≥18.5	2 3	5
Hou et al. (2021)	2021	China	12,964	65.17	64.83 ± 12.2	<25 ≥25	609 286	895
Kumral et al. (2021)	2021	Turkey	9285	56	66.2 ± 12.6	<30 ≥30	1355 843	2198
Kawase et al. (2017)	2017	Japan	1206	63	72.5 ± 11.4	<18.5 18.5–24.9 ≥25	15 129 52	196
Jingguang et al. (2016)	2016	China	100	77	67.2 ± 13.5	<25 ≥25	8 7	15
Andersen and Olsen (2015)	2015	Denmark	29,326	52	72.3	<18.5 18.5–24.9 25.0–29.9 ≥30	75 848 705 286	1914
Zhao et al. (2015)	2015	China	2360	61	Female: 66.4 Male: 62.7	<30 ≥30	—	668
Aiming. et al. (2014)	2014	China	510	51.18	≥18	<25 ≥25	77 121	198
Andersen and Olsen (2013)	2013	Denmark	28,382	54.6	71.5 ± 13.2	<18.5 18.5–24.9 25.0–29.9 ≥30.0	293 2480 1870 928	5571
Doehner et al. (2013)	2013	Germany	1472	53.5	59–84	<18.5 18.5–24.9 25–30 30–34.9 ≥35	1 37 34 6 4	82
Chen et al. (2012)	2012	China	620	54.5	72 ± 10.9	<25 ≥25	84 16	100
Ovbiagele et al. (2011)	2011	America	20,246	64	66.1 ± 8.6	<25 25–29.9 ≥30	600 778 427	1805
Vemmos et al. (2011)	2011	Denmark	2785	62	18–103	<25 25.0–29.9 ≥30	67 65 15	147
Olsen et al. (2008)	2008	Greece	21,884	52	≥40	<18.5 18.5–24.9 25.0–29.9 30.0–34.9 >35	271 2100 1516 548 166	4601

Abbreviation: BMI, body mass index.

### 3.3 Risk of Bias in Included Studies

The quality of the included studies was evaluated by the NOS scale, with a score of 5–8 indicating that 13 studies were high quality and 5 studies were moderate quality. See Table 2 for the specific evaluation results.

**TABLE 2:** Risk of bias assessment for included studies.

	Year	Selection of study populations				Comparability of the group whether to control for confounders	Result measurement			Total score
		Representativeness of the exposure group	Selection of the non‐exposing group	Determination of exposure factors	Demonstration that outcome of interest was not present at start of study		Independent evaluation by blind method	Follow‐up is long enough	Adequacy of follow‐up	
Hviid Hornnes et al. (2024)^a^	2024	1	1	1	1	2	1	1	1	9
Jingli et al. (2024)	2024	1	1	1	1	2	1	0	1	8
Chung et al. (2023)	2023	1	1	1	1	2	0	0	0	6
Ju et al. (2022)^a^	2022	1	1	0	0	2	1	0	0	5
Yan et al. (2021)^a^	2021	1	1	1	0	2	1	0	1	7
Hou et al. (2021)	2021	1	1	1	0	2	1	0	1	7
Kumral et al. (2021)^a^	2021	1	1	1	0	2	1	1	1	8
Kawase et al. (2017)^a^	2017	1	1	1	0	2	1	0	1	7
Jingguang et al. (2016)	2016	1	1	1	1	2	1	1	1	9
Andersen and Olsen (2015)	2015	1	1	1	0	2	0	0	1	7
Zhao et al. (2015)^a^	2015	1	1	1	0	2	1	0	1	7
Aiming. et al. (2014)	2014	1	1	1	1	2	1	1	1	9
Andersen and Olsen (2013)^a^	2013	1	1	1	0	2	1	1	1	8
Doehner et al. (2013)^a^	2013	1	1	1	0	2	0	0	1	6
Chen et al. (2012)	2012	1	1	1	0	2	1	0	0	6
Ovbiagele et al. (2011)^a^	2011	1	1	1	0	2	0	0	1	6
Vemmos et al. (2011)^a^	2011	1	1	1	0	2	1	0	1	7
Olsen et al. (2008)^a^	2008	1	1	1	0	2	1	1	1	8

^a^Studies were in meta‐analysis.

### 3.4 The Result of Meta‐Analysis

**FIGURE 2:**
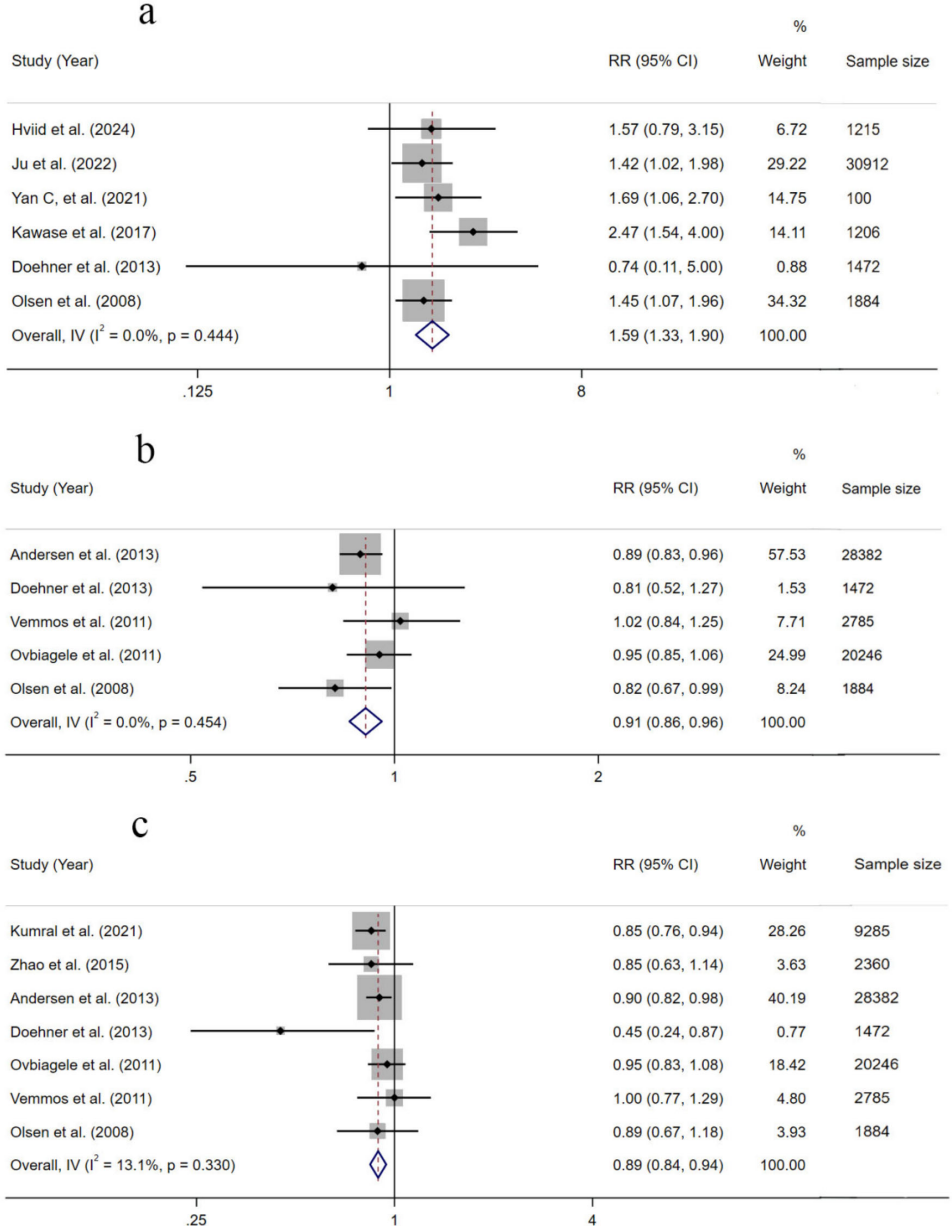
The result of meta‐analysis with normal weight as the reference group: (a) underweight group; (b) overweight group; (c) obesity group. CI, confidence interval; RR, relative risk.

All 18 studies reported the relationship between BMI and recurrence risk of stroke, but only 11 (Andersen and Olsen 2013; Doehner et al. 2013; Hviid Hornnes et al. 2024; Ju et al. 2022; Kawase et al. 2017; Kumral et al. 2021; Olsen et al. 2008; Ovbiagele et al. 2011; Vemmos et al. 2011; Yan et al. 2021; Zhao et al. 2015) were included in the meta‐analysis. Four (Aiming et al. 2014; C. Chen et al. 2012; Hou et al. 2021; Jingli et al. 2024) out of the remaining seven studies (Aiming et al. 2014; Andersen and Olsen 2015; C. Chen et al. 2012; Chung et al. 2023; Hou et al. 2021; Jingguang et al. 2016; Jingli et al. 2024) showed a correlation between BMI and stroke recurrence, whereas two studies (Chung et al. 2023; Jingguang et al. 2016) showed no correlation between BMI and stroke recurrence. Six studies combined underweight and normal‐weight patients into one group (BMI < 25 kg/m^2^) and overweight and obese patients into another group (BMI ≥ 25 kg/m^2^); it was not possible to extract further information about the recurrence risk of stroke in each BMI group. In addition, there was a high likelihood of double counting of data being present in a study (Andersen and Olsen 2015) that could collect the corresponding RR with 95% CI, which was from the same Danish cohort as the study of Andersen and Olsen (2013). After removing the study (Andersen and Olsen 2015), only 11 studies were included in the meta‐analysis. Among 11 studies (Andersen and Olsen 2013; Doehner et al. 2013; Hviid Hornnes et al. 2024; Ju et al. 2022; Kawase et al. 2017; Kumral et al. 2021; Olsen et al. 2008; Ovbiagele et al. 2011; Vemmos et al. 2011; Yan et al. 2021; Zhao et al. 2015), the normal BMI category in each study was used as a reference group, and we then compared it to other groups with different BMI ranges (underweight vs. normal BMI, overweight vs. normal BMI, and obese vs. normal BMI). The result of the meta‐analysis is presented in Figure 2.

### 3.5 Effect of Underweight and Normal BMI Categories on Recurrence Risk of Stroke

Six studies (Doehner et al. 2013; Hviid Hornnes et al. 2024; Ju et al. 2022; Kawase et al. 2017; Olsen et al. 2008; Yan et al. 2021) examined the association between underweight and recurrence risk of stroke. Results of the meta‐analysis showed that the recurrence risk of stroke in the underweight BMI group (BMI < 18.5 kg/m^2^) was 1.30 times higher compared to the normal BMI group (RR = 1.59, 95% CI 1.33–1.90, *I* ^2^ = 0%, *p = *0.444) (Figure 2a).

### 3.6 Effect of Overweight and Normal BMI Categories on Recurrence Risk of Stroke

Five studies (Andersen and Olsen 2013; Doehner et al. 2013; Olsen et al. 2008; Ovbiagele et al. 2011; Vemmos et al. 2011) examined the association between overweight and the recurrence risk of stroke. Results of the meta‐analysis showed that the risk of recurrent stroke decreased in the overweight BMI group (25 kg/m^2^ ≤ BMI ≤ 29.9 kg/m^2^) by 0.92 times that in the normal BMI group (RR = 0.91, 95% CI 0.86–0.96, *I* ^2^ = 0%, *p* = 0.454) (Figure 2b).

### 3.7 Effect of Obese and Normal BMI Categories on Recurrence Risk of Stroke

Seven studies (Andersen and Olsen 2013; Doehner et al. 2013; Kumral et al. 2021; Olsen et al. 2008; Ovbiagele et al. 2011; Vemmos et al. 2011; Zhao et al. 2015) examined the association between obesity and the recurrence risk of stroke. Results of the meta‐analysis showed that the risk of recurrent stroke decreased in the obese BMI group (BMI ≥ 30 kg/m^2^) by 0.89 times that in the normal BMI group (RR = 0.89, 95% CI 0.84–0.94, *I* ^2^ = 13.1%, *p *= 0.330) (Figure 2c).

### 3.8 The Result of Sensitivity Analysis

After excluding each study, there was no significant change in results, indicating that the results of the study were stable (Figure S1).

### 3.9 The Result of Dose–Response Analysis

**FIGURE 3:**
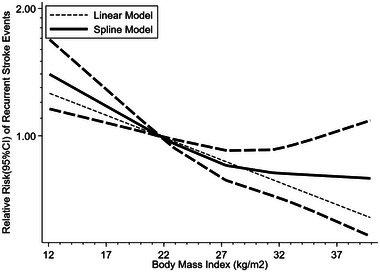
The result of dose–response analysis for the relationship between body mass index and recurrent stroke. The bold solid line and dashed line represent the estimated relative risk and its 95% confidence interval, respectively. The unbold dashed line represents the linear model. Referred to similar figures in Huang et al.’s study (2016). CI, confidence interval.

Six studies (Andersen and Olsen 2013; Doehner et al. 2013; Kawase et al. 2017; Olsen et al. 2008; Ovbiagele et al. 2011; Vemmos et al. 2011) were included in the dose–response analysis. Dose–response analysis showed no significant non‐linear relationship between BMI and the recurrence risk of stroke (*p* for non‐linearity = 0.104). The results of the linear trend show that for every unit increase in BMI, the recurrence risk of stroke decreases by 2% (RR = 0.98, 95% CI 0.96–0.99, *p *< 0.001) (Figure 3).

## 4 Discussion

The present study updated the study of Huang et al. (2016), demonstrating an inverse linear relationship between BMI and the recurrence risk of stroke. Although both studies revealed a consistent linear relationship, our study not only further identified a 2% reduction in stroke recurrence risk per one‐unit increase in BMI but also found that overweight and obese patients exhibited a significantly lower recurrence risk of stroke compared to underweight patients.

The results of the study indicate underweight is a risk factor for recurrent stroke, and the risk of recurrent stroke is increased in underweight patients by 1.30 times that of the normal BMI group. This may be due to BMI and the onset of stroke, which lead to various kinetics, such as a catabolic/anabolic imbalance and tissue wasting of both fat and muscle tissue (Scherbakov et al. 2011), and form a vicious cycle that worsens the nutritional status (Xue et al. 2008), subsequently causing a poor outcome. On the one hand, it may be that patients with underweight, whether malnourished or not, have mild low‐grade inflammation, and chronic inflammation may play a crucial role in the relationship between underweight and atherosclerosis (Ju et al. 2022). One study suggests that underweight is associated with impaired endothelial function, leading to a decrease in the bioavailability of nitric oxide, which can regulate vascular tension and inhibit platelet aggregation and adhesion (Higashi et al. 2002). On the other hand, low body weight can lead to a decrease in muscle mass, which is associated with excessive inflammation (Li et al. 2019), insulin resistance, and various metabolic disorders (Du et al. 2018), which may lead to myocardial infarction and stroke. Greater muscle mass is associated with better exercise ability and cardiovascular health, which may lead to a decreased risk of cardiovascular disease (Kwon et al. 2021). In addition, lower BMI can serve as an alternative marker for patient frailty, including malnutrition and non‐cardiovascular comorbidities, and frailty itself increases the risk of stroke (Adabag et al. 2018; Veronese et al. 2017).

Overweight and obesity are protective factors for stroke recurrence, with a decreased recurrence risk of stroke in overweight and obese patients of 0.92 and 0.89 times that in the normal BMI group, respectively. This conclusion is consistent with the “obesity paradox” found in previous studies (Andersen and Olsen 2013; Dehlendorff et al. 2014; Di Angelantonio et al. 2016; Vemmos et al. 2011). This may be related to overweight or obese patients having more metabolic reserves compared with normal‐weight patients, which helps them better cope with unbalanced catabolic status and impaired metabolic efficiency caused by stroke (Barba et al. 2015; Lavie et al. 2015). Furthermore, a soluble tissue necrosis factor receptor produced by adipose tissue can neutralize the deleterious effects of tumors on the development and progression of stroke (Ryu et al. 2011).

However, one study (Kumral et al. 2021) suggests that obesity is associated with an increased risk of recurrent stroke compared to non‐obese individuals, which may be related to several mechanisms in obese people, such as stress‐related neuroendocrine autonomic nerve activation, proinflammatory cytokines, an increased load of oxygen‐free radicals, and systemic hormone imbalance (Oesch et al. 2017; Scherbakov et al. 2011). Obesity can significantly aggravate the occurrence and development of atherosclerosis. On the one hand, adipocytes produce leptin, plasminogen activator inhibitor 1 (PAI‐1), adiponectin, tumor necrosis factor (TNF‐α), and other physiologically active substances that are directly involved in the formation of inflammation and atherosclerosis. On the other hand, obesity is one of the important risk factors for diabetes, hypertension, and hyperlipidemia, which will promote arteriosclerosis and may increase the recurrence risk of stroke (Wang et al. 2022).

Although we have explained the relationship between obesity and stroke recurrence, there is still controversy, possibly due to the heterogeneity of the study subjects, differences in sample size, and inconsistent BMI criteria. Further study is needed to explain the potential mechanisms leading to the obesity paradox in stroke recurrence patients.

Given the findings of this study, individual BMI categories should be considered when developing strategies to prevent the recurrence risk of stroke. Besides, to raise public awareness of the importance of healthy eating and weight management, public health policies should enhance healthy lifestyle promotion. In the future, prospective cohort studies with large sample sizes related to BMI and recurrent risk of stroke can be conducted in people of different ages or with different diseases while also exploring the impact of different types of obesity on stroke outcome or the impact of BMI on recurrence of different types of stroke so as to form personalized health management plans.

## 5 Strengths and Limitations

Several strengths exist in this study. First, compared with only 5 original studies included in Huang et al.’s (2016) meta‐analysis, this study included 11 original studies for meta‐analysis and provided a more detailed explanation of the relationship between BMI and the recurrence risk of stroke. In addition, the recent new original studies on the relationship between BMI and stroke recurrence were included, and most of the included studies had a large sample size. Finally, the literature included in this study was of good quality, with a high hierarchy of evidence. A total of 13 studies were of high quality, and 5 were of medium quality.

However, there are still some limitations that need to be addressed in this study. First, the retrieval language was confined to English or Chinese. Thus, publications in other languages may have been missed. Second, generalizability of the population may need to be further explored in future studies. Finally, the findings of the study are limited by the availability of detailed data, which may restrict generalizability. Future studies should collect more comprehensive data to strengthen the conclusion drawn from this study.

## 6 Conclusion

To sum up, the results of the current study showed a negative linear relationship between BMI and the recurrence risk of stroke, with each increased unit of BMI reducing the recurrence risk of stroke by 2%. Furthermore, BMI category is associated with the recurrence risk of stroke, strengthening the view of the obesity paradox that underweight is a risk factor for stroke recurrence, whereas overweight and obesity are protective factors for stroke recurrence. These findings inform future relevant studies in clinical decision‐making and secondary prevention of stroke and suggest that appropriate weight management, including nutrition and rehabilitation, is particularly important for patients with stroke.

## Author Contributions

**Qiuxia Qian**: conceptualization, methodology, formal analysis, data curation, writing–review and editing, writing–original draft. **Yuting Zhao**: conceptualization, methodology, writing–original draft, writing–review and editing, formal analysis, data curation. **Xin Fan**: methodology, formal analysis, data curation. **Jialu Li**: methodology, formal analysis, data curation. **Jianxun Cao**: methodology, formal analysis, data curation. **Mengyu Yang**: methodology, formal analysis, data curation. **Longchun Hua**: data curation, formal analysis. **Xingxia Zhang**: formal analysis, data curation. **Ailing Yang**: formal analysis, data curation. **Fengwa Zhang**: methodology, writing–review and editing, project administration, supervision. **Yuxia Ma**: methodology, writing–review and editing, supervision, project administration.

## Ethics Statement

The authors have nothing to report.

## Consent

The authors have nothing to report.

## Conflicts of Interest

The authors declare no conflicts of interest.

## Permission to Reproduce Material From Other Sources

Not applicable.

## Peer Review

The peer review history for this article is available at https://publons.com/publon/10.1002/brb3.70550.

## Supporting information



Table S1 PubMed search strategy.
Figure S1 Sensitivity analysis on the recurrence risk of stroke associated with different BMI categories with normal weight as the reference group: (a) underweight group; (b) overweight group; (c) obesity group.

## Data Availability

Research data are not shared.
