# Supplementary material for: The Relationship Between Body Mass Index and Recurrence Risk of Stroke: A Systematic Review and Dose–Response Meta‑Analysis
Source: Brain Behav. 2025 May 29;15(6):e70550. doi: 10.1002/brb3.70550 (PMC12120265; doi:10.1002/brb3.70550)
Supplement: Supplementary file 1 — Table S1 PubMed search strategy. Figure S1 Sensitivity analysis on the recurrence risk of stroke associated with different BMI categories with normal weight as the reference group: (a) underweight group; (b) overweight group; (c) obesity group. [file BRB3-15-e70550-s001.DOCX]

## Supplementary Table 1 PubMed search strategy.

|  | **Search Terms** |
| --- | --- |
| #1 | "Stroke"[MeSH Terms] |
| #2 | "Stroke"[Title/Abstract] OR "Strokes"[Title/Abstract] OR "Apoplexy"[Title/Abstract] OR "acute stroke*"[Title/Abstract] OR "acute cerebrovascular accident*"[Title/Abstract] OR "brain vascular accident*"[Title/Abstract] OR "cerebral stroke*"[Title/Abstract] OR "cerebrovascular accident*"[Title/Abstract] OR "cerebrovascular apoplexy"[Title/Abstract] OR "cerebrovascular stroke*"[Title/Abstract] OR "ischemic stroke"[Title/Abstract] OR "cerebral infarction"[Title/Abstract] OR "brain infarction"[Title/Abstract] OR "cerebral hemorrhage"[Title/Abstract] OR "cerebrovascular events"[Title/Abstract] |
| #3 | #1 or #2 |
| #4 | "Body Mass Index"[MeSH Terms] |
| #5 | "body mass index"[Title/Abstract] OR "BMI"[Title/Abstract] OR "quetelet index"[Title/Abstract] OR "quetelet s index"[Title/Abstract] OR "quetelets index"[Title/Abstract] OR "Thinness"[Title/Abstract] OR "Underweight"[Title/Abstract] OR "Leanness"[Title/Abstract] OR "ideal body weight"[Title/Abstract] OR "ideal body weight*"[Title/Abstract] OR "normal weight*"[Title/Abstract] OR "normal body weight*"[Title/Abstract] OR "ideal body mass*"[Title/Abstract] OR "ideal body mass index"[Title/Abstract] OR "Overweight"[Title/Abstract] OR "over weight*"[Title/Abstract] OR "overweight*"[Title/Abstract] OR "Obesity"[Title/Abstract] OR "obese"[Title/Abstract] OR "fat"[Title/Abstract] OR "adipos*"[Title/Abstract] OR "obes*"[Title/Abstract] OR "weight loss"[Title/Abstract] |
| #6 | #4 or #5 |
| #7 | "Recurrence"[MeSH Terms] |
| #8 | "Recurrence"[Title/Abstract] OR "recurrence rate"[Title/Abstract] OR "recrudescence"[Title/Abstract] OR "Relapse"[Title/Abstract] OR "relapse rate"[Title/Abstract] OR "Reappear"[Title/Abstract] OR "recidivation"[Title/Abstract] |
| #9 | #7 or #8 |
| #10 | #3 and #6 and #9 |

**
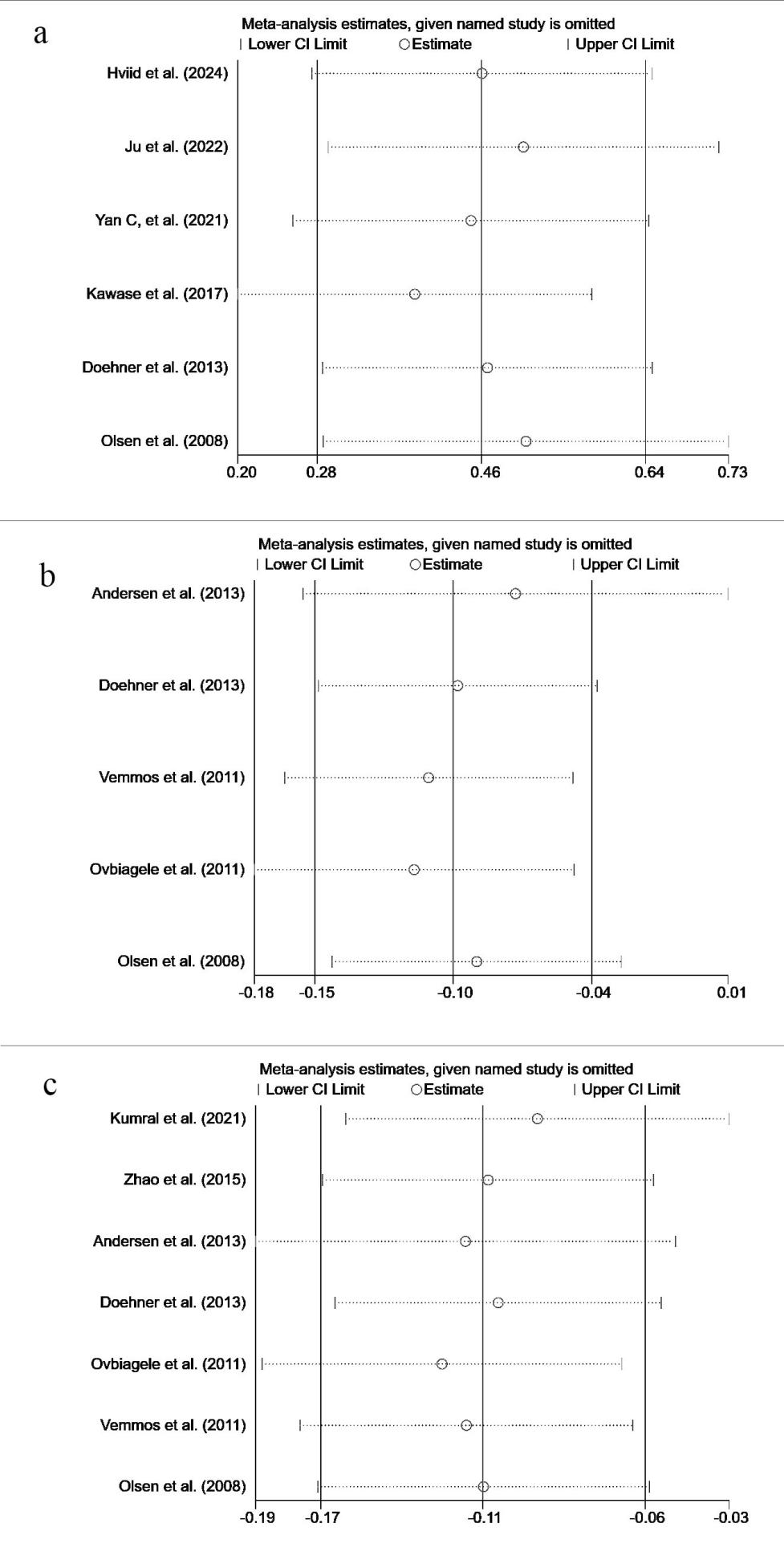
**

**Supplementary Fig. 1 Sensitivity analysis on the recurrence risk of stroke associated with different BMI categories with normal weight as the reference group.** a, underweight group; b, overweight group; c, obesity group.
